# SOCIAL DIFFERENCES IN ALLOSTATIC LOAD TRAJECTORIES ACROSS ADULTHOOD

**DOI:** 10.1093/geroni/igac059.1637

**Published:** 2022-12-20

**Authors:** Christine Walsh, Yang Yang, Daniel Belsky, Sarah Mills, Marissa Hall, Shelley Golden

**Affiliations:** Carolina Population Center, University of North Carolina, Chapel Hill, North Carolina, United States; University of North Carolina at Chapel Hill, Chapel Hill, North Carolina, United States; Mailman School of Public Health, Columbia University, New York City, New York, United States; University of North Carolina at Chapel Hill, Chapel Hill, North Carolina, United States; University of North Carolina at Chapel Hill, Chapel Hill, North Carolina, United States; University of North Carolina at Chapel Hill, Chapel HIll, North Carolina, United States

## Abstract

Chronic psychosocial stress has been indicated as a potentially important driver of U.S. health disparities. Allostatic load (AL) is an indicator of cumulative biological risk or “wear and tear” on the body resulting from repeated adaptations to life stressors. A population-level examination of AL may be particularly useful in understanding the intermediate physiological dysregulation that underlies social inequalities in health. No study has yet documented changes in allostatic load over a wide range of ages and intersecting sociodemographic groups. To address this gap, we pooled biomarker data from three U.S. longitudinal cohort studies (National Longitudinal Study of Adolescent to Adult Health, Midlife Development in the U.S., and the Health and Retirement Survey) to create an integrated dataset covering individuals ages 24 to over 95 from several birth cohorts. We conducted growth curve models to examine associations of AL trajectories with cohort and multiple dimensions of social identity. We found more recently born cohorts were associated with higher mean levels in AL as compared to earlier-born cohorts. Net of cohort, education, and other covariates, Black men and women and Hispanic men had significantly higher AL than their White counterparts. These differences were present in early adulthood and persisted at all ages. Hispanic women experienced the largest increases in AL with age, surpassing nearly all other groups at older ages. These findings contribute new knowledge about the temporal dynamics and social patterning of AL as individuals age, particularly intersecting stress exposures as an underlying and enduring source of health inequity.

